# The relevance of nerve mobility on function and activity in children with Cerebral Palsy

**DOI:** 10.1186/s12883-016-0715-z

**Published:** 2016-10-07

**Authors:** Petra Marsico, Amir Tal-Akabi, Hubertus J. A. van Hedel

**Affiliations:** 1 Paediatric Rehab Research Group, Rehabilitation Centre for Children and Adolescents, University Children’s Hospital Zurich, Mühlebergstrasse 104, CH-8910 Affoltern am Albis, Switzerland; 2 Bern University of Applied Sciences, Health, Murtenstrasse 10, CH-3008 Bern, Switzerland; 3 Children’s Research Centre (CRC), University Children’s Hospital Zurich, Steinwiessstrasse 75, CH-8032 Zürich, Switzerland

**Keywords:** Neurodynamic, Neuromobilization, Neural tissue, Movement limitations, Range of Movement, Muscle strength, Gross motor function, Participation

## Abstract

**Background:**

In children with cerebral palsy (CP), stiffness, caused by contractile and non-contractile structures, can influence motor performance. This study sought to determine whether the nerve mobility had a relevant impact on motor performance in children with CP. We hypothesized that a positive Straight Leg Raise (SLR) test, as well as smaller SLR hip angle, would relate to lower leg muscle strength, reduced motor capacity and less motor performance in children with CP.

**Methods:**

We applied a cross-sectional analysis on data including SLR, leg muscle strength, Gross Motor Function Measure (GMFM-66) and number of activity counts during daily life from thirty children with CP (6–18 years). We performed receiver operating characteristics and correlation analyses.

**Results:**

Positive SLR test could distinguish well between children with low versus high muscle strength and GMFM-66 scores. The SLR hip angle correlated significant with the level of disability and with muscle strength. The correlation with the GMFM-66 and the activity counts was fair.

**Conclusion:**

This study suggests that neural restriction of SLR is higher on functional and activity outcome than the measured SLR hip range of motion. Further studies should investigate weather improving nerve mobility can lead to an amelioration of function in children with CP.

## Background

The movement of neural tissue relative to its surrounding structures is necessary for normal physiological functioning [1]. Impaired movement or elasticity of the nervous system can provoke pain or restriction of movement. Indeed, different studies demonstrated the existence of nerve gliding during limb movements [2]. In rats, even a mild nerve compression without major axonal loss is sufficient to induce a local and remote immune-inflammatory response [3]. In humans, similar observations were made in patients suffering from neuro-orthopaedic dysfunctions, such as carpal tunnel syndrome [4, 5]. In adult patients with lesions of the central nervous system, the neural structures influenced the motor performance, observed as a relationship between lack of nerve mobility and lower extremity muscle strength or reduced motor performance [6]. Shacklock suggested using the term “neurodynamics” when discussing interaction between nervous system and mechanics and physiological function [7]. Neurodynamic techniques combined with proprioceptive neuromuscular facilitation were more effective in reducing sensory deficits than traditional therapy consisting of home exercises, balance and locomotor training, in adult patients recovering from stroke [8]. In two studies with small sample sizes, the myoelectric activity of spasticity was reduced following neural mobilization in patients with stroke [9, 10]. Based on these promising studies in adults, we propose applying these techniques also to children and adolescents with congenital or postnatal lesions of the central nervous system, such as in children with cerebral palsy (CP).

Characteristic symptoms of CP like muscle weakness, resistance against movement such as stiffness or spasticity and deformity of the joints could contribute to restrictions in daily movement, such as a shorter step length during walking or difficulty in climbing stairs [11]. When investigating the factors underlying stiffness or deformities, both contractile and, non-contractile components of the muscle and surrounding tissues were in the focus [12]. For example, while the sarcomeres (i.e. a contractile component) of the individuals with CP were increased in length, and in contrary, an accumulation of collagen (i.e. non-contractile component) indicated stiffness in the hamstring muscles [13]. Still, it is not quite clear in which impairments, such as muscle weakness, stiffness or joint deformities influence the restriction in children with CP [14]. Based on the promising results by applying neurodynamic techniques in adult patients, we suggest that the neural structures could be an additional factor influencing movement. However, research about neural structures and their influence on functional limitations and recovery in children with neurological disorders is scarce. One study showed a relationship between a neurodynamic test evaluating mobility of the sciatic nerve (slump test) and trunk control in children with CP of varying severity [15]. In a retrospective study, a fair correlation could be shown between ‘straight leg raise’ (SLR), hip angle or range of motion and maximal knee extension during gait (*r* = 0.34) [16]. However, the authors did not include sensitizing manoeuvres to observe if the neural structures specifically were the source of restricting the movement of the SLR hip range of motion.

Therefore, the main goal of this study was to observe the discriminative ability of the SLR in case of functional and activity status of these children. We hypothesized that children with a positive SLR (sciatic nerve as limiting factor of the movement) or reduced SLR hip range of motion tend to have lower muscle strength, reduced functional capacity and lower level of spontaneous motor performance than children with a negative SLR or higher range of motion, respectively.

## Methods

### Study design

We used a prospective cross-sectional observational study design to determine the relationship between the SLR and various functional outcomes.

### Participants

Children were recruited from the in- and out-patient setting of the Rehabilitation Centre for Children and Adolescents of the University Children’s Hospital Zurich in Affoltern am Albis. Inclusion criteria were: diagnosis of CP, aged 6 to 18 years, the ability to communicate pain, follow simple instructions and to extend the knee against resistance. All children agreed to participate. Participants were excluded from the study if they had an arthrodesis of the ankle joint, a flexion contracture of more than 5° in the knee joint or less than 80° hip flexion with the knee flexed. Further exclusion criteria were CP with primary athetosis (rather than spastic), surgery of the lower limbs or back within the preceding twelve months and imposed medical restriction for weight bearing. Twenty-one children, who participated in a previous study investigating reliability of the SLR test, did also participate in this study [17]. The goal was to recruit 30 children.

### Measures

**Fig. 1:**
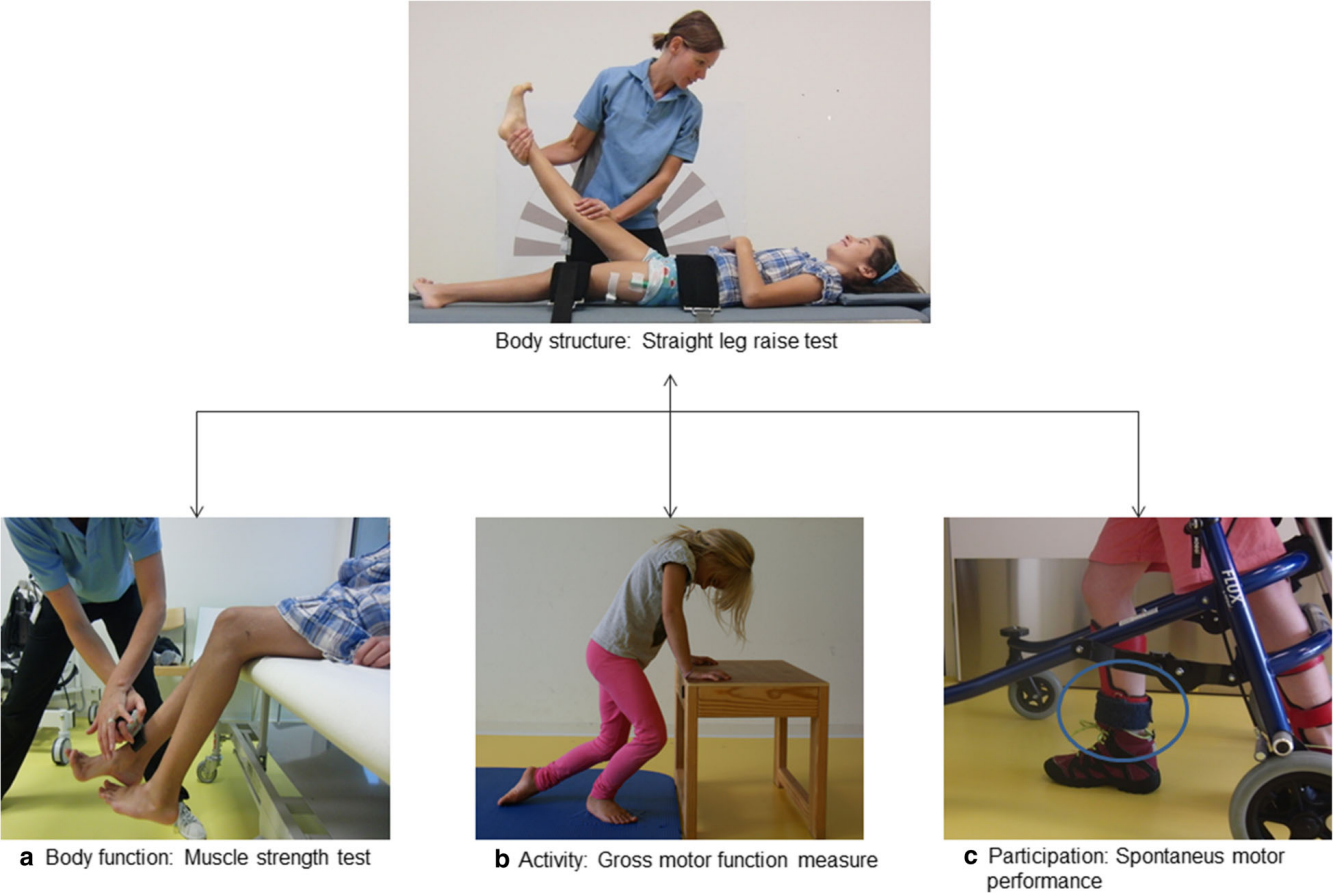
Comparing the Straight Leg Raise test with the functional measurements. Straight Leg Raise (SLR) performance (above), **a** measure of knee extensor strength with a hand-held dynamometer (ICF: body function), **b** exercise of the gross motor function measure-66 (ICF: activity), **c** accelerometer worn on the right ankle to measure the daily activity (ICF: participation)

Measures were selected to cover all domains according to the International Classification of Functioning, disabilities and health (ICF) [18]. The bio-psycho-social model of the ICF describes the health condition or disorder on three domains: body structure and function, activity and participation (Fig. 1).

### Body structure: straight leg raise

In this study, the SLR was applied to observe mobility of the sciatic nerve on the body structure level. An electrogoniometer (Noraxon, USA. Inc., Scottsdale, AZ, USA) was used to measure hip range of motion. Sensors were placed on the lateral shank (on a line between the trochanter major and epicondyles lateral) and the lateral pelvis. In the neutral position of the hip joint, the electrogoniometer was calibrated for the starting position to 0°. SLR testing was performed first for the right leg with the child rested in supine with a 2 cm thick foam as head support. The pelvis and the contralateral leg were stabilized with straps. To differentiate whether the SLR hip range of motion was limited due to muscle length or due to lack of a neural mobility, structural differentiation maneuvers were used. For this structural differentiation, two test performances were applied in the form of sensitizing movements, first with initial dorsal flexion and second with initial neck flexion. The test was applied according to the test description of a previous study by the authors [17]. The leg was moved slowly, at approximately 5° per second, as this minimized the influence of (velocity-dependent) spasticity. The test was rated as positive if the SLR hip range of motion increased by at least 5° when ankle dorsal flexion (sensitizing movement 1) and neck flexion (sensitizing movement 2) were released. A positive SLR test indicates the sciatic nerve as limiting factor of the movement. For the correlation analysis, the maximal SLR hip range of motion of both sides was selected.

### Body function: muscle strength test

On the body function level knee extensor muscle strength was measured with a hand-held dynamometer (microFET2, Hoggan Health Industries, Salt Lake City, UT, USA). For these measurements, the child sat on a bench with dangling lower legs (Fig. 1a). The child was allowed to use its hands for support without grasping on the edge of the bench, the knee was in 20° flexed position. If the children could not extend the leg until 20° knee flexion, due to insufficient strength, the strength was measured at the highest possible knee extension position. Knee extensor strength was evaluated bilaterally with the “make method”, i.e. the child had to push against the hand-held dynamometer [19]. The knee was in 20° flexed position. This approach has a high reliability (ICC = 0.83–0.95) in children with CP [20]. Each leg was measured 3 times, with a 2-min break between the sessions. Then, the mean of the three repeated tests was calculated for further analysis.

### Activity: gross motor function measure

On the activity level the Gross Motor Function Measure (GMFM-66), a validated measurement to score the functional capacity in children with CP, was conducted [21]. The GMFM-66 is composed of gross motor activities lying, rolling, sitting, crawling, kneeling, standing, walking, jumping and running (Fig. 1b). The gross motor ability estimator (GMAE) converts the results of the GMFM-66 into percentages, with 100 % representing the maximally achievable score of the test.

### Participation: spontaneous motor performance

On the participation level, a 3D accelerometer, the Actiwatch 2® (Actiwatch, Cambridge Neurotechnology Ltd, Cambridge UK) evaluated the daily motor activity of the child (Fig. 1c). Accelerometry is a widely used and validated method for assessing physical activity in children with CP [22]. The children wore the Actiwatch® on the right ankle for at least 48 h on weekdays. The Actiwatch was removed only for bathing or showering. The epoch length was set to one minute and the unit was counts per minutes (cpm). More cpm indicated a higher amount of motor performance. Sleeping periods were automatically excluded by the program itself. Externally induced activity, such as robot-assisted treadmill training or passive mobilisation, was excluded for the analysis of the Actiwatch®. Out-patients received an activity diary to report their activities during the day while wearing the Actiwatch®. For in-patients, the rehabilitation day schedule provided information about their daily activity. The Actiwatch® data were collected and analysed using the Actigraph software (Respironics Actiware 5, Version 5.59.0015; Cambridge Neurotechnology Ltd, Cambridge UK) to determine the activity score.

### Assessment procedure

All measurements took place within a week. The tests were performed in a quiet room. Two one hour sessions were planned to apply the different tests. In the first session, the first author (paediatric physiotherapist and trained in neurodynamic) performed the SLR test followed by the muscle strength measurement. In the second session, the GMFM was performed by one of four trained physiotherapists of our centre. We further recorded age, bodyweight, height, and the diagnoses including the disability severity levels as expressed by the Gross Motor Function Classification System (GMFCS).

### Data analysis

Statistical analyses were performed with SPSS (IBM SPSS Statistics 19, Chicago, IL). All variables were visually inspected for normal distribution (histogram) and skewness and kurtosis were analysed. Means and Standard Deviation (SD) or median and Interquartile Range (IQR) for each measurement were calculated.

To estimate if a positive SLR related to less muscle strength, a lower GMFM-66 and reduced activity score, Receiver Operating Characteristics (ROC) were applied [23]. For each measure, corresponding cut-off levels were calculated, based on the highest Youden-Index (=sensitivity + specificity-1) [24]. The Area Under the Curve (AUC) was presented as an indicator of the accuracy of the ROC-analysis. The AUC was considered acceptable (0.7–0.8), excellent (0.8–0.9) or outstanding (≥0.9) [25]. Point-biserial correlations were calculated between the dichotomous SLR rating (positive or negative) and the three measurements.

For the correlations between the SLR hip range of motion and the three measurements Pearson correlations (r) or Spearman correlations (r_s_), depending on the normality of distribution, were used.

To evaluate if the SLR rating (positive or negative) correlates with the measured SLR hip range of motion again point-biserial correlations were calculated.

For the interpretation of the correlation coefficient, the following benchmarks were used: little relationship (0–0.24), a fair degree of relationship (0.25–0.49), a moderate to good relationship (0.50–0.74), and a very good to excellent relationship (0.75–1.00) [26].

## Results

### Participants

We included 30 children aged between 6y 5months and 17y (mean age 10y 4months [SD 3y 3months]; 13 girls, 17 boys) in the study. The recruiting period did last two years. Eighteen of them were in-patients, and 12 were out-patients. Three children had a selective dorsal rhizotomy at least four years ago. The other 27 participants had no previous orthopaedic surgery. Patients’ characteristics and results of the applied measurements, according to their level of disability as expressed by the GMFCS level, are presented in Table 1.

**Table 1:** Number of participants and result of the applied measures presented for the Gross Motor Function Classification System

Classification	Frequency (n)	Age (years) mean(SD)	Straight leg raise rating (+/-)		Straight leg raise (°) mean(SD)		Muscle strength (N) median (IQR)^a^		GMFM-66 (%) mean(SD)	Actiwatch (cpm) mean(SD)
			left	right	left	right	left	right		
GMFCS, level I	6	11(2)	2/4	2/4	50(12)	46(18)	154(95)	112(38)	92(12)	515(195)
GMFCS, level II	6	14(3)	3/3	2/4	42(9)	42(9)	89(32)	106(22)	77(5)	402(195)
GMFCS, level III	9	10(3)	7/2	7/2	37(10)	36(11)	53(48)	53(45)	61(12)	200(96)
GMFCS, level IV	6	11(3)	6/0	6/0	40(13)	38(8)	14(48)	8(55)	36(9)	86(80)
GMFCS, level V	3	11(1)	3/0	3/0	22(8)	25(2)	0(0)	0(0)	34(11)	208(183)
Total	30	10(3)	21/9	20/10	40(13)	38(12)	75(22)	72(19)	62(4)	279(39)

*Abbreviations*: *SD* Standard Deviation, *GMFCS* Gross Motor Function Classification System, *N* Newton, *cpm* counts per minute, *GMFM-66* Gross Motor Function Measure-66, *IQR* Interquartile Range

^a^Comment. In the group of GMFCS-Level 3 and 4 two children could not perform the muscle strength test (*n* = 28)

The SLR test could be measured in all children. Three measurements of hip range of motion had to be performed manually, due to technical problems with the electrogoniometer. The same landmarks were used as with the sensors of the electrogoniometer. The mean SLR hip range of motion on the right side was 38° SD12° and on the left side 40° SD13°.

In two children, the muscle strength could not be measured accurately, once due to involuntary movements (child with GMFCS level IV) and once due to cognition and attention deficits (child with GMFCS level III). These tests were excluded pairwise for the corresponding calculation. The median knee extensor strength for the right leg was 72 N (IQR = 91 N, *n* = 28) and of the left leg 75 N (IQR = 71 N, *n* = 28). The mean GMFM-66 score was 62 % (SD = 4 %; *n* = 30). The children had worn the Actiwatch® for a mean duration of 71 ± 16 h during day time over 3 to 5 days, with a mean of 279 cpm (SD = 39 cpm; *n* = 30). There was a very good correlation between the GMFM-66 and the activity score (*r* = 0.83; *p* < 0.001). Also, the muscle strength (mean right left side) correlated significantly with the GMFM-66 (*r*_s_ =0.84; *p* < 0.001) and with the activity score (*r*_s_ = 0.67; *p* < 0.001).

### Relationship between the SLR and the functional measurements

**Fig. 2:**
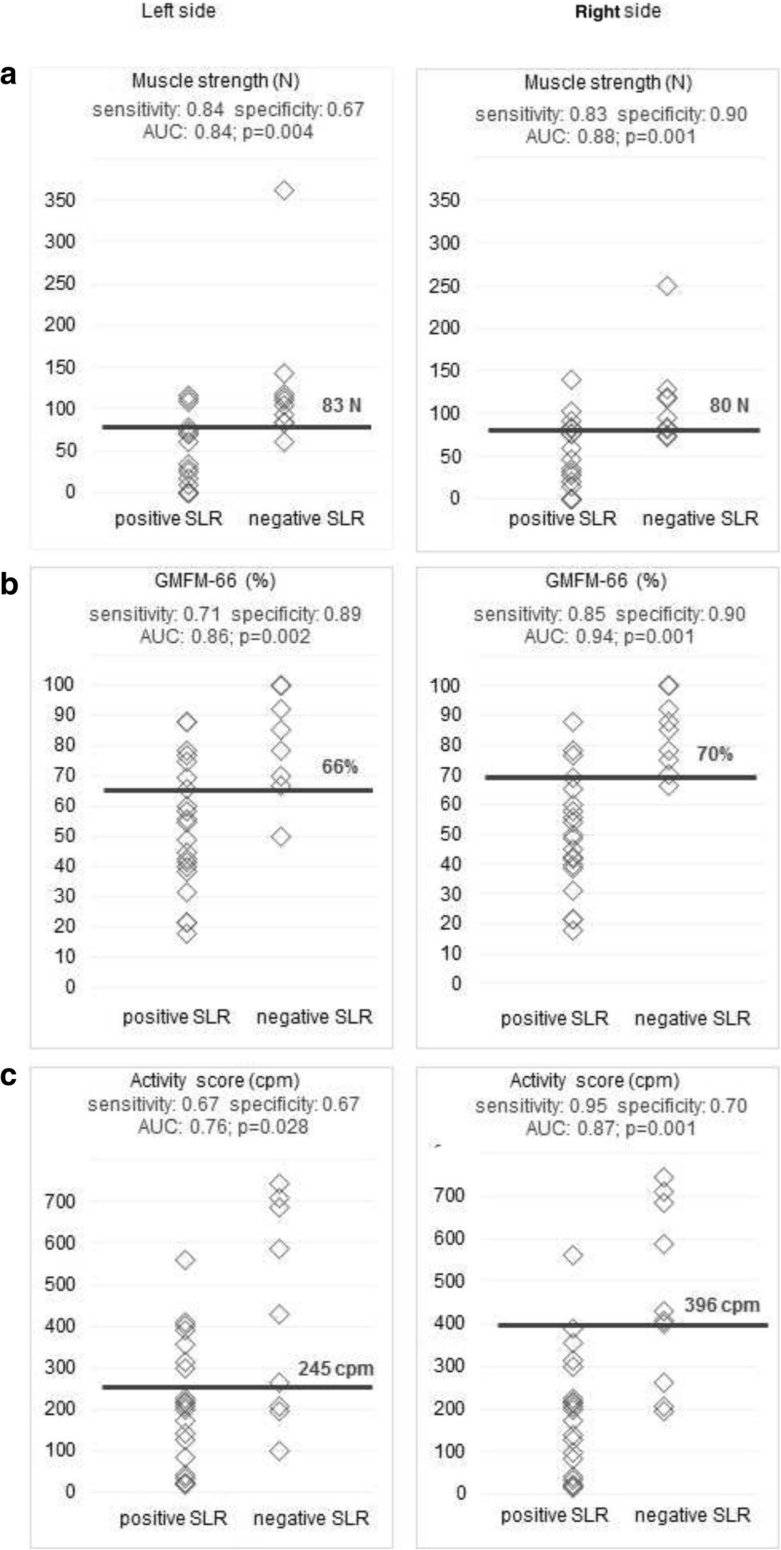
Discriminative ability of a positive and negative Straight Leg Raise test. Cut off values with sensitivity and specificity, area under the curve (AUC) for all three functional measurements: **a** muscle strength in newton (N), **b** Gross Motor Function Measure-66 and **c** activity score in Counts per Minute (cpm)

**Fig. 3:**
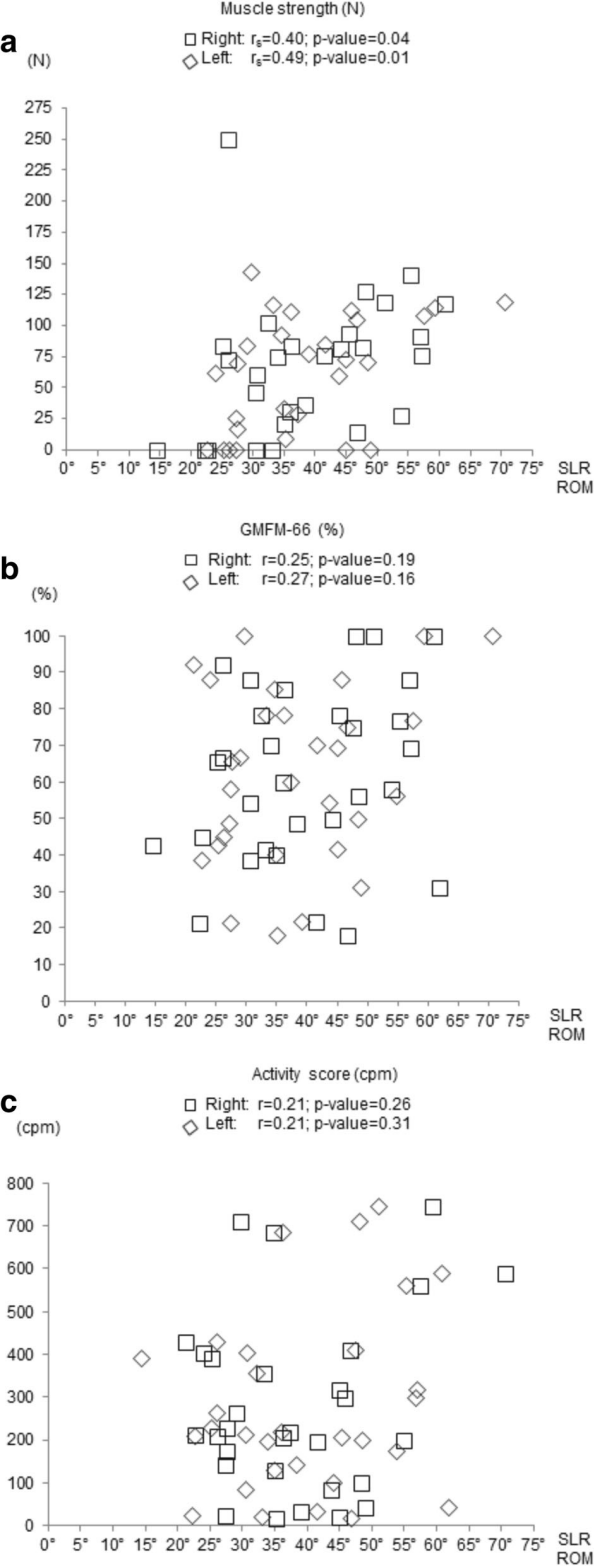
Correlations between Straight Leg Raise hip range of motion and outcome measures. Spearmen correlation (r_s_) between Straight Leg Raise (SLR) hip Range of Motion (ROM) and **a** muscle strength measured in newton (N); Pearson correlation (r) between **b** SLR hip ROM and ‘gross motor function measure-66’ (GMFM-66) and **c** SLR hip ROM and activity score in Counts per Minute (cpm)

In general, a positive SLR test could distinguish well between children with higher and lower functional status (Fig. 2). The SLR differentiated best between children with high versus low GMFM-66 scores, followed by muscle strength and, finally, activity counts. Point-biserial correlations showed moderate to good relationship for all three measurements and the dichotomous SLR rating. The results were for knee extensor strength for the left leg: 0.58 correlation, *p* = 0.001; right leg: 0.56 correlation, *p* = 0.002, for the GMFM-66 left leg: 0.56 correlation, *p* = 0.001; right leg: 0.70 correlation, *p* < 0.001, and for the activity counts left leg: 0.50 correlations, *p* = 0.006; right leg; 0.63 correlation, *p* < 0.001. The correlations between the SLR hip range of motion and muscle strength were fair and significant (Fig. 3a). Little to fair degree of relationship were found between the SLR hip range of motion and GMFM-66 (Fig. 3b, c). The correlation (r_s_) between the GMFCS levels and the SLR hip ROM was for the right side *r*_s_ = -0.37; *p* = 0.047 and for the left side *r*_s_ = -0.51; *p* = 0.006. This negative correlation indicates that children with a higher level of disability tend to have a reduction in SLR hip range of motion.

The correlation between the SLR rating (positive or negative) and the SLR hip range of motion was little (right side *r* = 0.075; *p* = 0.69; left sde *r* = 0.082; *p* = 0.66).

## Discussion

The aim of this study was to determine the relationship between SLR test results and functional measures in a group of children with CP. We hypothesised that a positive SLR can be a predictor for reduced motor function, capacity and performance. A good correlation between the SLR hip range of motion and the functional measurements and disability level was expected. To our knowledge, this is the first study investigated the relationship between the mobility of the neural structures and functional outcomes.

A positive SLR (nerve as limiting factor) or negative SLR (other structures such as muscle length) was able to distinguish between children with higher and lower motor function, capacity and performance with acceptable sensitivity, and specificity, and excellent high areas under the curves. For example, a positive SLR differentiated best between children with a better versus poorer motor score, when the GMFM-66 cut-off value was set at 66–70 %.

The design of our study allows us only to hypothesize about the underlying mechanisms. As we know that more severely disabled children move less in daily life, they are more at risk for limitations in movements and mobility [27]. As a consequence of the reduced movements the peripheral nerves, could loss a part of their adaptive ability to limb movement. Typically movements over more than one joint were limited by involvement of limitations in nerve mobility, such as the straight leg raise. This again constrains motor ability and might lead to a vicious circle.

Even if the correlations between the GMFCS levels and the SLR hip range of motion where significant, the correlation with the other three measures and the SLR hip range of motion was little to fair. The findings were analogue to the study by McMulkin et al. [16]. Indeed, some children with a mild hemiplegic CP (GMFCS level I) with consequently good scores in GMFM-66 and high activity levels showed a small SLR hip range of motion on the more affected side. The moderate to good correlations of the SLR rating (positive or negative) and the three functional measurements was a better estimator for functional mobility in children with CP than the SLR hip range of motion, which showed only little to fair correlations. This indicates that the lack of neural mobility might be more important compared to the length of the hamstrings muscles, limiting SLR hip range of motion, when investigating mechanisms underlying restrictions in functional mobility. Indeed, the little correlation between SLR rating, and hip range of motion suggests an independent between these measures.

In addition, age-specific changes in SLR hip range of motion, such as reduced SLR hip range of motion during years of growth could also influence our findings [28]. We assume that age did not play an important role in our study, as age did not correlate with SLR hip range of motion (right leg: *r* = 0.065; *p* = 0.73; left leg: -0.014, *p* = 0.94).

### Limitations of the study

The study has several limitations: The population is very heterogeneous concerning the participants’ level of functional limitations and disability. Nevertheless, to observe a first possible relationship it was our intention to cover the majority of children with CP (excluded children with primary athetoid CP). Three of the six children with unilateral spastic CP had over 10° less SLR hip range of motion on the more effected side. Therefore, we would recommend testing this group separately when observing movement limitation due to nerve mobility.

### Clinical implications

Functional limitations in children with CP are often reflected in restrictions of mobility due to resistance of a specific structure or reduced force generation [14]. To distinguish between the sources of restrictions while performing activities of daily life, such like walking, climbing stairs or putting on shoes, physiotherapists include different assessments in their clinical reasoning process. For example, if the goal of a child with crouch gait is to increase step length or decrease knee flexion in initial contact, the therapist should be aware that this child needs to fulfil the structural requirements to perform this movement. From a functional point of view, when walking and flexing a leg forward during the late swing phase (with extended knee and dorsal flexed ankle joint), typically developing children and adolescents need about 37° (SD: 6.7°) of hip flexion at initial contact during walking, with around 5° dorsiflexion and 5° knee flexion while the other leg is in hip extension [29]. Since this position is comparable to the SLR test (sensitizing movement 1) including dorsiflexion, the SLR could be a confirmation of resistance due to neurodynamic components. Therefore, we suggest interpreting the SLR in relation to function or activity for the clinical reasoning process.

## Conclusion

This study is a first step towards research of neurodynamic testing and treatment in the neuro-paediatric field. The results of this study showed a relationship between mobility related functions, activities, and participation and restrictions of nerve mobility in children with CP. Therefore, integration of neurodynamic assessments to the clinical reasoning process might provide an additional view to observe and treat mobility limitations in children with CP. Further research is needed to get more insight into the causality between neural structures and mobility limitations in this group of children.

## Abbreviations

SLR: Straight leg raise
CP: Cerebral palsy
GMFM: Gross Motor Function Measure
GMFCS: Gross Motor Function Classification System
ICF: International Classification of Functioning Disability and Health
SD: Standard Deviation
IQR: Interquartile Range
ROC: Receiver Operating Characteristics
AUC: Area under the Curve
CPM: Counts per Minutes
